# A Redox-Nucleophilic Dual-Reactable Probe for Highly Selective and Sensitive Detection of H_2_S: Synthesis, Spectra and Bioimaging

**DOI:** 10.1038/srep30148

**Published:** 2016-07-21

**Authors:** Changyu Zhang, Runyu Wang, Longhuai Cheng, Bingjie Li, Zhen Xi, Long Yi

**Affiliations:** 1 State Key Laboratory of Organic-Inorganic Composites, Beijing University of Chemical Technology (BUCT), Beijing, China; 2 State Key Laboratory of Elemento-Organic Chemistry, Department of Chemical Biology, National Engineering Research Center of Pesticide (Tianjin), Nankai University, Tianjin, China; 3 Collaborative Innovation Center of Chemical Science and Engineering, Nankai University, Tianjin, China

## Abstract

Hydrogen sulfide (H_2_S) is an important signalling molecule with multiple biological functions. The reported H_2_S fluorescent probes are majorly based on redox or nucleophilic reactions. The combination usage of both redox and nucleophilic reactions could improve the probe’s selectivity, sensitivity and stability. Herein we report a new dual-reactable probe with yellow turn-on fluorescence for H_2_S detection. The sensing mechanism of the dual-reactable probe was based on thiolysis of NBD (7-nitro-1,2,3-benzoxadiazole) amine (a nucleophilic reaction) and reduction of azide to amine (a redox reaction). Compared with its corresponding single-reactable probes, the dual-reactable probe has higher selectivity and fluorescence turn-on fold with magnitude of multiplication from that of each single-reactable probe. The highly selective and sensitive properties enabled the dual-reactable probe as a useful tool for efficiently sensing H_2_S in aqueous buffer and in living cells.

Hydrogen sulfide (H_2_S) is an important endogenous signalling molecule with multiple biological functions^1^,^2^,^3^,^4^,^5^,^6^,^7^. H_2_S could be enzymatically produced by three distinctive pathways including cystathionine β-synthase (CBS), cystathionine γ-lyase (CSE) and 3-mercaptopyruvate sulfurtransferase (3-MPST)/cysteine aminotransferase (CAT) in different organs and tissues^3^,^4^. Studies have shown that the H_2_S level *in vivo* is correlated with numerous diseases, including the symptoms of Alzheimer’s disease, Down syndrome, diabetes and liver cirrhosis^1^,^8^. Despite H_2_S has been recognized to be linked to numerous physiological and pathological processes, many of its underlying molecular events *in vivo* remain largely unknown. Therefore, it presents significant research value to develop efficient methods for detection of H_2_S in living biological systems.

Compared with traditional methods^9^,^10^,^11^,^12^, fluorescent probes should be excellent tools for *in situ* monitoring H_2_S in biological samples because of their non-destructive sensing of bio-targets with readily available detection^13^,^14^,^15^,^16^,^17^,^18^,^19^,^20^,^21^,^22^,^23^,^24^,^25^,^26^,^27^,^28^,^29^,^30^,^31^,^32^,^33^,^34^,^35^,^36^,^37^,^38^,^39^,^40^,^41^,^42^. Organic reactions including H_2_S-mediated reduction^14^,^15^,^16^,^17^,^18^,^19^,^20^,^21^,^22^,^23^,^24^,^25^,^26^,^27^,^28^, nucleophilic addition/substitution^29^,^30^,^31^,^32^,^33^, and dual-nucleophilic addition/substitution^34^,^35^,^36^,^37^,^38^,^39^ were employed for development of H_2_S fluorescent probes. Though the great success of these fluorescent probes, we still need to develop probes with higher selectivity and sensitivity for detection of biological H_2_S in living systems. We have proposed a dual-reactive and dual-quenching strategy for improvement of probe’s selectivity and sensitivity, respectively^40^,^41^,^42^. However, the combination usage of both redox reaction and nucleophilic reaction for H_2_S probes was rarely explored^42^. Furthermore, in our previous work for dual-reactable probes^40^,^41^,^42^, we did not prepare “exact” control probes (single-reactable probes with the same fluorophore and reaction group as that of the dual-reactable probe) for comparable studies, which was insufficient for understanding the properties of dual-reactable probes. Herein, we report a dual-reactable probe **1** based on 1,8-naphthalimide as fluorophore (Fig. 1) for highly selective and sensitive detection of H_2_S in living cells. The two single-reactable control probes **2** and **3** were also prepared, which revealed that the improved turn-on fold and selectivity of the dual-reactable probe could be magnitude of multiplication from that of the two single-reactable probes **2** and **3**.

## Results and Discussion

To obtain H_2_S fluorescent probes with higher selectivity, we decided to develop dual-reactable H_2_S probes based on both redox and nucleophilic reactions. However, our previous probes were based on multi-step organic synthesis and coumarin fluorophores with relatively short emission^42^. The reduction of aromatic azide to amine is the most used redox reaction for H_2_S probe^13^,^14^,^15^,^16^,^17^,^18^,^19^,^20^,^21^,^22^,^23^,^24^. The nucleophilic reaction of thiolysis of NBD (7-nitro-1,2,3-benzoxadiazole) amine have been explored by us to develop H_2_S probes^31^. In this work, we used the reduction of aromatic azide and thiolysis of NBD amine for development of a new dual-reactable fluorescence probe **1**. The synthesis of **1** is straightforward from commerically available reagents. Moreover, both NBD and azide moieties could quench fluorescence of the naphthalimide fluorophore in **1** through fluorescence resonance energy transfer (FRET) and intramolecular charge transfer (ICT) effects, respectively.

The synthesis of **1** was achieved by coupling reaction of single-reactable probe **3** and NBD-Cl. Probe **3** was prepared from commerically available reagents 4-bromo-1,8-naphthalic anhydride, sodium azide and 4-amino-1-Boc-piperidine. The facile and economic synthesis is important for the wide use of such type of the dual-reactable probe. For control study, probe **2** was prepared by a five-step synthesis (see ESI). Probes **1**–**3** were well characterized by ^1^H NMR, ^13^C NMR and HRMS (see ESI).

**Figure 2:**
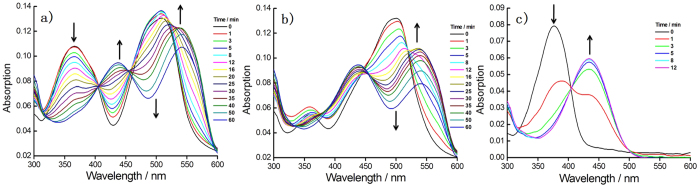
Absorption spectra revealed that probe 1 underwent both redox and nucleophilic reactions in the presence of H_2_S. Time-dependent absorption spectra of **1** (**a**) or **2** (**b**) or **3** (**c**) in the presence of H_2_S. Probes were 10 μM. For (**a**,**b**), H_2_S were 2 mM; for (**c**), H_2_S was 1 mM.

The absorption spectra of the probes **1**–**3** were further examined for understanding the mechanism (Fig. 2). The dual-reactable probe **1** exhibited absorbance peaks at 365 nm and 506 nm, which were assigned to azide naphthalimide and NBD moieties, respectively. Upon treatment with H_2_S, a time-dependent decrease at 365 nm and an increase at 435 nm with an isosbestic point at 405 nm were observed (Fig. 2a), due to the reduction of azide to amine; because the control probe **3** exhibited the similar change with the same isosbestic point (Fig. 2c). The NBD absorbance for **1** or **2** in the presence of H_2_S displayed a decrease absorbance at around 500 nm and an increase at about 535 nm, respectively, due to thiolylsis of NBD amine. The NBD-based probe **2** showed slower reaction rate toward H_2_S than that of the azide-based probe **3** (Fig. S1), and the reaction kinetics of the dual-quenching probe **1** was mainly decided by the property of the relative slow reaction site. In summary, the absorption change of single-reactable probes toward H_2_S showed similar response with that of the one-reactive site from the dual-reactable probe, implying that the dual-reactable probe could undergo two orthogonal reactions with H_2_S simultaneously.

**Figure 3:**
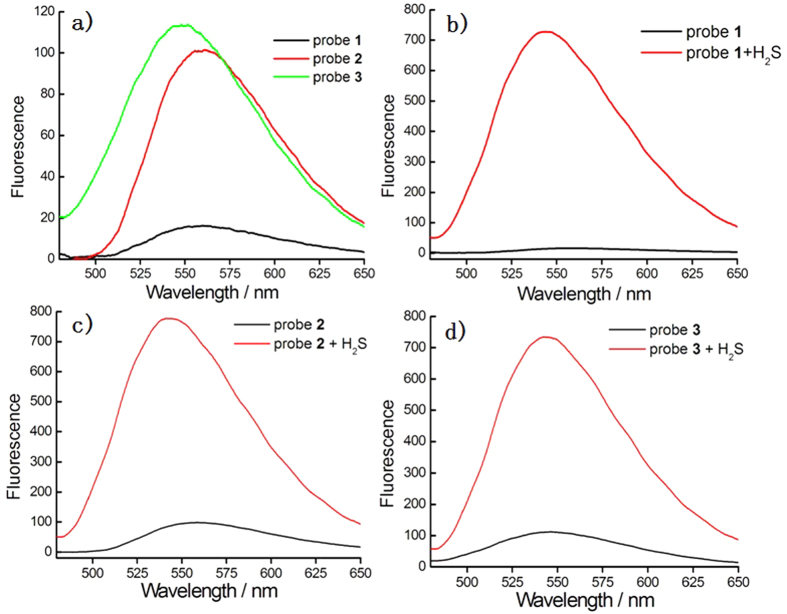
The dual-reactable probe 1 gave higher fluorescent turn-on response toward H_2_S than that of single-reactable probes 2 and 3. (**a**) The fluorescence spectra of pure probes **1–3** (1 μM) upon excitation at 425 nm in PBS buffer (pH 7.4). (**b**) Fluorescence response of **1** (1 μM) toward H_2_S (500 μM) for 60 min. (**c**) Fluorescence response of **2** (1 μM) toward H_2_S (500 μM) for 60 min. (**d**) Fluorescence response of **3** (1 μM) toward H_2_S (200 μM) for 30 min. Slits for all spectra: 5/10 nm (excitation/emission).

As shown in Fig. 3, emission spectra of probes **1**–**3** were checked in the absence or presence of H_2_S in PBS (pH 7.4). As expected, the naphthalimide emission was heavily quenched for probe **1** due to FRET-ICT dual-quenching effects. While single-reactable probes **2** and **3** showed relative strong fluorescence under similar test conditions. These results implied that the combined usage of FRET-ICT dual-quenching effects is more efficient than that of any FRET or ICT single-quenching effect. After reacting with H_2_S, the probe **1** showed significantly turn-on fluorescent response at 540 nm, with off-on response up to ca. 54.8 fold for **1**. The *off-on* response of **2** or **3** upon H_2_S treatment was ca. 9.5 fold or 6.6 fold, either of which was much smaller than that of **1**, while the turn-on fold of **1** could be determined by multiplication of each turn-on fold of single-reactable probes **2** and **3**. Therefore, we can draw the conclusion that the fluorescence turn-on fold upon reacting with H_2_S for dual-reactable probe is indeed greatly increased via dual-quenching effect.

**Figure 4:**
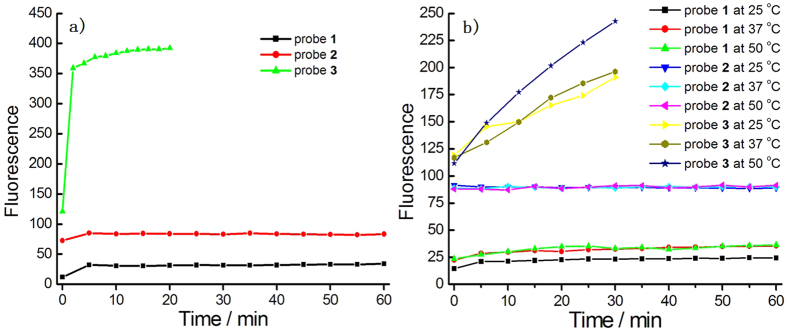
Investigation of probes’s stability by fluorescence. Fluorescence intensity at 540 nm versus time of probes **1**–**3** (1 μM) in PBS buffer under a UV lamp (365 nm, 16 W) (**a**) or not (**b**).

In next tests, the dual- and single-reactable probes were incubated in PBS buffer for the thermo- and photo-stability tests (Fig. 4). The results indicated that the fluorescence increase of dual-reactable probe **1** was almost negligible even under UV light for 1 h, while the single-reactable azide-based probe showed an obvious fluorescent increase (Fig. 4a). Even without UV light, single-reactable probe **3** showed a certain fluorescent increase at different temperature while the dual-reactable probe **1** had much better stability (Fig. 4b). These results further indicated the advance of the dual-reactable probe.

A major challenge for H_2_S detection in biological systems is to develop a selective probe that exhibits distinctive response to H_2_S over millimolar biothiols and other reactive sulfur species. To design a highly selective H_2_S probe, we used dual-reactable groups on one fluorophore; and the reactable group is also the quenching group. If a competitor can react with 10% probe in nucleophilc reaction site or with 20% in redox site, the maximal turn-on effect for a dual-reaction quenched fluorescent probe is only about 2% (10%^*^20%). Such a dual-reactable strategy should increase the probe’s selective response between H_2_S and the competitor.

**Figure 5:**
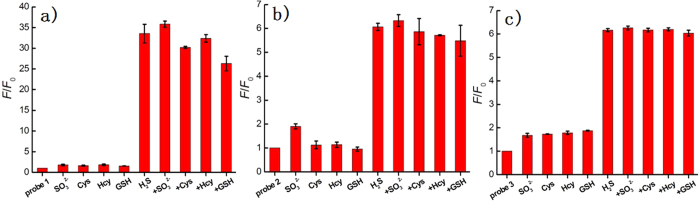
The dual-reactable probe 1 is more selective toward H_2_S than that of single-reactable probes 2 and 3. Relative emission intensities at 540 nm (excitation at 425 nm) of probe **1** (**a**) or **2** (**b**) or **3** (**c**) in the presence of test species in PBS (pH 7.4). The test lanes: SO_3_^2−^ (200 μM), Cys (1 mM), Hcy (1 mM), GSH (5 mM), H_2_S (200 μM), H_2_S (200 μM) + SO_3_^2−^ (200 μM), H_2_S (200 μM) + Cys (1 mM), H_2_S (200 μM) + Hcy (1 mM), H_2_S (200 μM) + GSH (5 mM).

Probes **1**–**3** were incubated with various biological-related species in PBS and the maximal emission change was measured accordingly (Fig. 5 and S2). The tested species included biothiols (GSH, 5 mM; Cys, 1 mM; Hcy, 1 mM), reactive oxygen species (H_2_O_2_, ClO^−^), reactive sulfur species (SO_4_^2−^, S_2_O_3_^2−^, SO_3_^2−^), anions (NO_2_^−^, N_3_^−^) and cations (Zn^2+^, Fe^3+^). For dual-reactable probe **1**, fluorescence intensity enhancement for any tested molecule in PBS (pH 7.4) was almost negligible except H_2_S. While for single-reactable probes **2** and **3**, SO_3_^2−^ or biothiols showed a certain fluorescence response. The dual-reactable probe **1** was further tested for its competitive selectivity over millimolar biothols and SO_3_^2−^ anion. The results indicated that probe **1** has higher selectivity than that of the single-reactable probes and is highly selective toward H_2_S over other biologically relevant species. These results indicated that the selectivity of **1** could be determined by multiplication of selectivity of single-reactable probes **2** and **3**, which is indeed greatly improved via dual-reactable effect.

To obtain the detection limit for **1**, the fluorescence intensity change was closely monitored by addition of various concentrations of H_2_S into the probe (Fig. S3a). The fluorescence intensity at 540 nm was linearly related to the concentrations of H_2_S from 5 to 40 μM, and the detection limit for **1** was calculated to be 0.9 μM by using the 3σ/k method^12^. The results implied that probe **1** is sensitive enough toward H_2_S in buffer solution. The detection limit of probes **2** or **3** was determined to be 2.5 μM or 2.6 μM, respectively, which was lower than that of the dual-reactable probe **1** (Fig. S3). We also investigated the fluorescence response of probe **1** to H_2_S under different pH values (Fig. S4). Results indicated that the probe **1** can function over a wide range of pH from 6.0 to 8.5, and the best response range is within pH 7.4–8.5. However, in the case of weak acidic conditions (pH < 7.0), the fluorescence turn-on signal is marginally lower, which was commonly observed in nucleophilic-reaction probes^29^,^30^,^31^,^32^,^33^.

**Figure 6:**
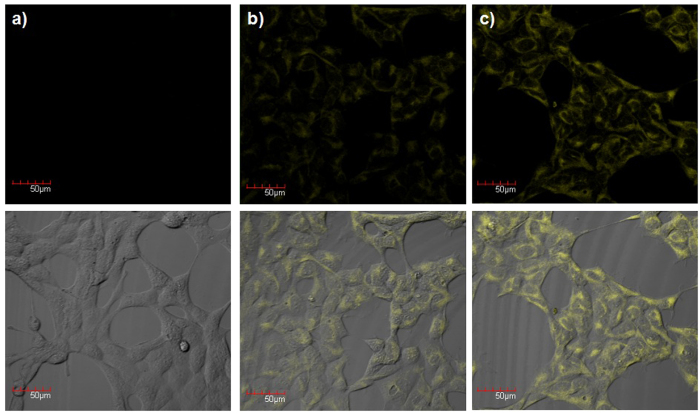
Confocal microscopy images of exogenous H_2_S in living cells using probe 1. HEK293 cells were incubated with (**a**) **1** (5 μM) for 30 min, (**b,c**) **1** (5 μM) for 30 min, washed by PBS buffer, and then Na_2_S ((**b**) 50 μM; (**c**) 200 μM) for 60 min. The merge images between fluorescent and bright-field images are below. Scale bar, 50 μm.

To test the biological applicability of the dual-reactable probe **1**, we examined whether **1** can be used to detect exogenous H_2_S in living cells (Fig. 6). HEK293A cells were treated with probe **1** and then washed with PBS to remove excess **1**. The **1**-loaded cells were incubated with Na_2_S (50 or 200 μM) and subsequently imaged using a confocal fluorescence microscopy. The addition of both probe **1** and H_2_S resulted in an obvious yellow fluorescence while the cells treated with only probe **1** did not show fluorescence. Merge images show that cells retained good morphology after incubation with **1** (Fig. 6), which suggested the good biocompatibility of **1**. The cytotoxicity of the probe **1** was further evaluated by MTT assay (Fig. S6), which did not show obvious cytotoxicity at 1–10 μM range. These studies implied that **1** is cell-permeable and can image intracellular H_2_S in living cells.

**Figure 7:**
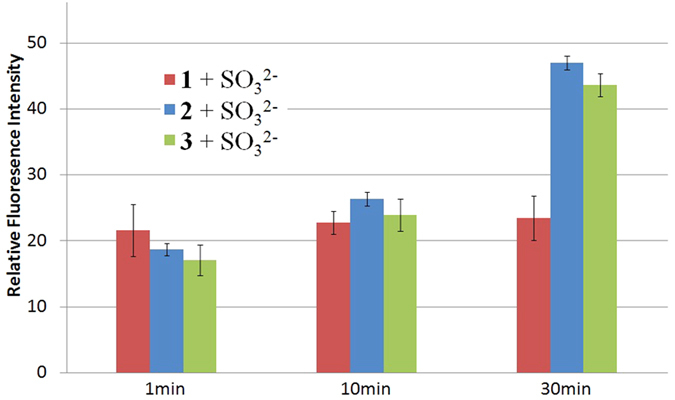
The dual-reactable probe 1 is more selective than that of single-reactable probes 2 and 3 in bioimaging. The average fluorescence of confocal microscopy images for probes **1**–**3** (5 μM) after the addition of SO_3_^2−^ anions (250 μM) for 1 min, 10 min and 30 min.

Finally, we compared the selectivity for bioimaging of dual-reactable probe **1** and its corresponding control probes **2** and **3** in living cells. Firstly, cells were treated with probes and then washed with PBS buffer to remove excess probes. The selectivity experiments indicated that sulfite anions could react with both single-reactable probes **2** and **3**, and therefore sulfite anions were added in the probe-loaded cells. The time-dependent fluorescent images and intensities (Fig. 7 and S5) indicated that **1**-loaded cells kept instant fluorescence while **2**- and **3**-loaded cells showed increased fluorescence. These preliminary results implied that the dual-reactable probe **1** could be more selective in bioimaging than that of single-reactable probes **2** and **3**.

## Conclusion

A new redox-nucleophilic dual-reactable fluorescent probe based on naphthalimide as fluorophore was developed for H_2_S detection in aqueous buffer and in living cells, which showed higher selectivity, stability and fluorescent turn-on fold than that of single-reactable probes **2** and **3**. The “exact” control probes of **1** revealed that the improved turn-on fold and selectivity of the dual-reactable probe could be magnitude of multiplication from that of the two single-reactable probes **2** and **3**. Furthermore, the dual-reactable probe **1** could be successfully used to image exogenous H_2_S in living cells selectively and efficiently. Our results further imply that using such redox-nucleophilic dual-reactable strategy could be general for preparation of highly selective and sensitive H_2_S probes for various biological applications.

## Methods

### Synthesis of 3

4-Azido-1,8-naphthalic anhydride^24^ (4.83 g, 20.2 mmol) and 4-amino-1-Boc-piperidine (3.64 g, 18.2 mmol) were dissolved in ethanol (200 ml), the mixture was heated to reflux with stirring overnight. The reaction was monitored by TLC on pre-coated silica plates. After cooling down to room temperature, the reaction mixture was added with ice water to obtain a yellow precipitate, which was collected by vacuum filtration and washed with ice water. The resulting residue was subjected to column chromatography on silica (0.5% MeOH in CH_2_Cl_2_), yielding a yellow solid **6** (2.6 g, 31%). *R*_f_ (5% MeOH in CH_2_Cl_2_), 0.6. ^1^H NMR (400 MHz, CDCl_3_) *δ* 8.57 (d, *J* = 7.3 Hz, 1H), 8.52 (d, *J* = 8.0 Hz, 1H), 8.38 (d, *J* = 8.4 Hz, 1H), 7.74–7.68 (m, 1H), 7.43 (d, *J* = 8.0 Hz, 1H), 5.21–5.09 (m, 1H), 4.29 (d, *J* = 26.9 Hz, 2H), 2.84 (s, 2H), 2.78–2.66 (m, 2H), 1.66 (d, *J* = 12.7 Hz, 2H), 1.48 (s, 9H); ^13^C NMR (101 MHz, CDCl_3_) *δ* 164.4, 164.0, 154.8, 143.4, 132.3, 131.8, 129.2, 128.7, 127.0, 124.3, 123.0, 119.3, 114.8, 79.6, 77.5, 77.2, 76.8, 51.9, 28.6, 28.4. Probe **3** was obtained by treatment of **6** (960 mg, 2.3 mmol) with TFA:CH_2_Cl_2_ (1:1) solution at room temperature, which was removed under reduced pressure. The resulting residue was dissolved by ethyl acetate, distilled with saturated NH_4_Cl solution and dried finally to obtain a yellow powder. ^1^H NMR (400 MHz, *d*_*6*_-DMSO) *δ* 8.50 (d, *J* = 7.3 Hz, 1H), 8.44 (d, *J* = 8.0 Hz, 1H), 8.38 (d, *J* = 8.4 Hz, 1H), 7.84 (t, *J* = 7.9Hz, 1H), 7.72 (d, *J* = 8.0Hz, 1H), 5.18–5.07 (m, 1 H), 3.34 (d, *J* = 11.9 Hz, 2 H), 2.98 (t, *J* = 12.2 Hz, 2H), 2.82–2.70 (m, 2H), 1.81 (d, *J* = 11.9 Hz, 2H); ^13^C NMR (101 MHz, *d*_*6*_-DMSO) *δ* 163.7, 163.2, 142.7, 131.7, 131.6, 128.4, 128.3, 127.4, 123.4, 122.5, 118.5, 116.0, 26.0. HRMS: calcd for [M + H]^+^, 322.1299; found 322.1302.

### Synthesis of 1

To a solution of **3** (290 mg, 0.9 mmol) and 4-chloro-7-nitrobenzofurazan (360 mg, 1.8 mmol) in 10 ml anhydrous DMF, DIPEA (618 μl, 3.6 mmol) was added drop by drop. The reaction mixture was stirred at room temperature for 3 h and then poured into 120 ml ice water, which was distilled with CH_2_Cl_2_ and dried. The resulting residue was subjected to column chromatography on silica (0.5% MeOH in CH_2_Cl_2_), yielding a red solid **1** (414 mg, 95%). *R*_f_ (5% MeOH in CH_2_Cl_2_), 0.6. ^1^H NMR (400 MHz, CDCl_3_) *δ* 8.57 (d, *J* = 7.3 Hz, 1H), 8.52 (d, *J* = 8.0 Hz, 1H), 8.43 (d, *J* = 4.5 Hz, 1H), 8.41 (d, *J* = 3.9 Hz, 1H), 7.73 (t, *J* = 7.9 Hz, 1H), 7.45 (d, *J* = 8.0 Hz, 1H), 6.35 (d, *J* = 9.0 Hz, 1H), 5.58–5.45 (m, 1H), 5.00 (d, *J* = 9.1 Hz, 2H), 3.60 (t, *J* = 12.3 Hz, 2H), 3.05–2.89 (m, 2H), 2.03 (d, *J* = 10.0 Hz, 2H); ^13^C NMR (101 MHz, CDCl_3_) *δ* 164.4, 164.0, 145.0, 145.0, 144.9, 143.9, 135.5, 132.6, 132.1, 129.2, 129.1, 127.1, 124.3, 123.1, 122.6, 118.8, 114.9, 102.6, 50.1, 49.9, 28.1. HRMS: calcd for [M+H]^+^, 485.1316; found 485.1320.

### Procedure of fluorescence measurements

Fluorescence studies were carried out using F-280 spectrophotometer (Tianjin Gangdong Sci & Tech., Development. Co., Ltd). 1–1000 mM Stock solutions of Na_2_S in degassed PBS buffer were used as H_2_S source. Probes were diluted in PBS buffer (pH = 7.4, 50 mM, 30% DMSO) to afford the final concentration of 1–10 μM. For the selectivity experiment, different biologically relevant molecules (100 mM) were prepared as stock solutions in degassed PBS buffer. Appropriate amount of biologically relevant species were added to separate portions of the probe solution and mixed thoroughly. The reaction mixture was shaken uniformly before emission spectra were measured. For the time-course experiment, 1 μM probe in PBS buffer were added with 500 μM or 200 μM Na_2_S at room temperature, and the emission was measured at different time points. For the pH-dependent experiment, probe **1** (1 μM) and Na_2_S (200 μM) were incubated with PBS buffers at different pH values. All measurements were performed in a 3 ml corvette with 2 ml solution.

### Cell culture and Bioimaging

HEK-293 cells were cultured at 37 °C, 5% CO_2_ in DMEM/HIGH GLUCOSE (GIBCO) supplemented with 10% fetal bovine serum (FBS), 100 U/ml penicillin, 100 μg/ml streptomycin, and 4 mM L-glutamine. The cells were maintained in exponential growth, and then seeded in glass-bottom 35 mm plate at the density about 2 × 10^4^/well. Cells were passaged every 2–3 days and used between passages 3 and 10. Cells were imaged on a confocal microscope (Olympus FV1000 UPLSAPO40X) with a 40 × objective lens. Emission was collected at yellow channel (500–600 nm) with 405 nm excitation. All images were analyzed with Olympus FV1000-ASW.

## Additional Information

**How to cite this article**: Zhang, C. *et al*. A Redox-Nucleophilic Dual-Reactable Probe for Highly Selective and Sensitive Detection of H_2_S: Synthesis, Spectra and Bioimaging. *Sci. Rep.* **6**, 30148; doi: 10.1038/srep30148 (2016).

## Supplementary Material

Supplementary Information

## Figures and Tables

**Figure 1:**
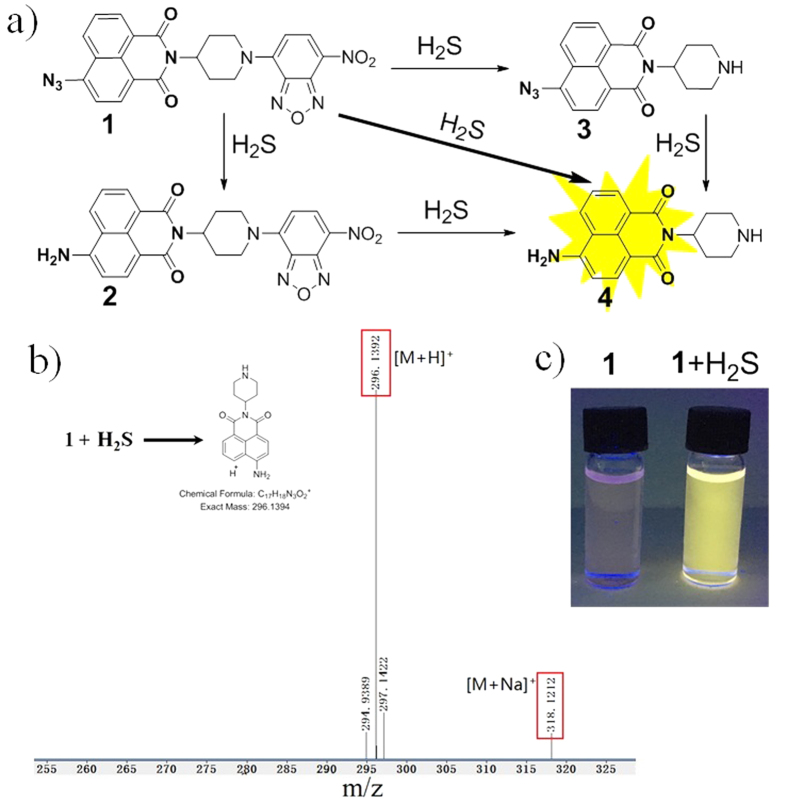
Reaction of the dual-reactable probe toward H_2_S. (**a**) Structure of a dual-reactable probe **1** and its reaction with H_2_S to give single-reactable probes **2** and **3** and the fluorophore **4**. (**b**) High resolution mass spectrum for the reaction solution of probe **1** and H_2_S revealed the production of **4**. (**c**) Photo of probe **1** and its reaction with H_2_S under 365 nm UV lamp.
