# Supplementary material for: A Redox-Nucleophilic Dual-Reactable Probe for Highly Selective and Sensitive Detection of H2S: Synthesis, Spectra and Bioimaging
Source: Sci Rep. 2016 Jul 21;6:30148. doi: 10.1038/srep30148 (PMC4954965; doi:10.1038/srep30148)
Supplement: Supplementary Information [file srep30148-s1.pdf]

Electronic Supplementary Information for

A Redox-Nucleophilic Dual-Reactable Probe for Highly Selective and Sensitive Detection of H_2S: Synthesis, Spectra and Bioimaging

Changyu Zhang, †[1] Runyu Wang, †[2,3] Longhuai Cheng,[2,3] Bingjie Li,[1] Zhen Xi[2,3] & Long Yi[1,3]

[1] State Key Laboratory of Organic-Inorganic Composites, Beijing University of Chemical Technology (BUCT), Beijing, China.
[2] State Key Laboratory of Elemento-Organic Chemistry, Department of Chemical Biology, Nankai University, Tianjin, China.
[3] Collaborative Innovation Center of Chemical Science and Engineering (Tianjin)
Correspondence and requests for materials should be addressed to L.Y. (e-mail: yilong@mail.buct.edu.cn) or Z.X. (zhenxi@nankai.edu.cn).
†These authors contributed equally to this work.

Table of contents

General information

Synthesis and Characterisation of compounds

Absorption analyses

Determination of the detection limit

Calculation of fluorescence turn-on fold

MTT assay

Figure S1. Time-dependent fluorescent spectra and dynamic simulation of probes 1-3

Figure S2. Selectivity experiments of probes 1-3

Figure S3. The detection limit of probes 1-3

Figure S4. Effects of pH on probe 1 in PBS buffer

Figure S5. Confocal microscopy images of SO_3^{2-} in living cells using probes 1-3

Figure S6. The cytotoxicity of probe 1 evaluated by the MTT assay

Figure S7-21. NMR and HRMS spectra of compounds

General information

All chemicals and solvents used for synthesis were purchased from commercial suppliers and applied directly in the experiments without further purification. The progress of the reaction was monitored by TLC on pre-coated silica plates (Merck 60F-254, 250 μm in thickness), and spots were visualized by basic $KMnO_4$, UV light or iodine. Merck silica gel 60 (100-200 mesh) was used for general column chromatography purification. ^{1}H NMR and ^{13}C NMR spectra were recorded on a Bruker 400 spectrometer. Chemical shifts are reported in parts per million relative to internal standard tetramethylsilane ($Si(CH_3)_4$ = 0.00 ppm) or residual solvent peaks ($CDCl_3$ = 7.26 ppm; d_6-DMSO = 2.5 ppm). ^{1}H NMR coupling constants (*J*) are reported in Hertz (Hz), and multiplicity is indicated as the following: s (singlet), d (doublet), t (triplet), dd (doublet of doublets), m (multiple). HRMS were obtained on an Agilent 6540 UHD Accurate-Mass Q-TOFLC/MS or Varian 7.0 T FTICR-MS.

Synthesis and Characterisation of compounds

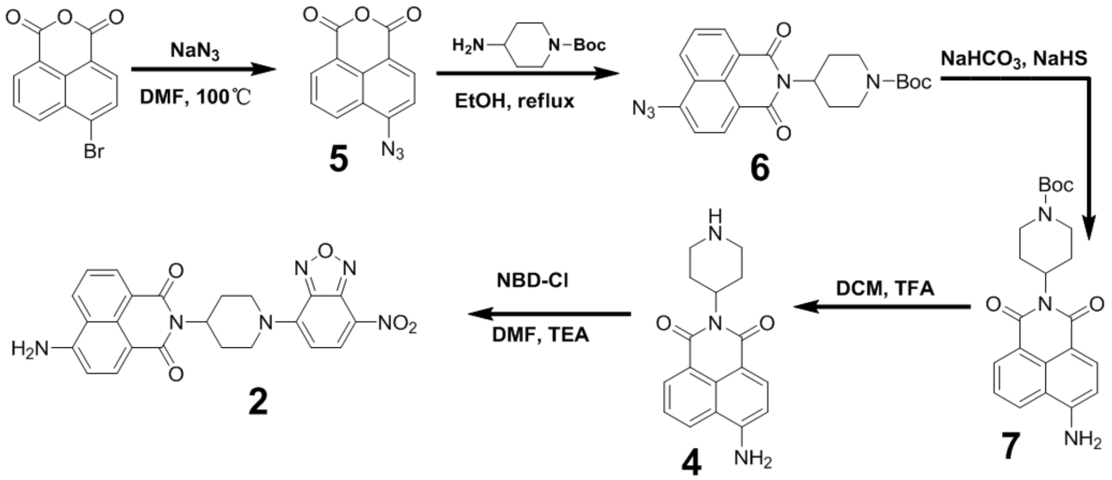

Synthesis of **5**. To a solution of 4-bromo-1,8-naphthalic anhydride (13.0 g, 47.3 mmol) in DMF (50 ml), NaN_3 (3.5 g, 53.8 mmol) in 1 ml water was added under N_2 protection, then the mixture was heated to 100 ℃ for 1 h. The solution was poured into a breaker containing ice water, forming a yellow precipitate, which was filtered and rinsed with water and ethanol. The product was dissolved by CH_2Cl_2, then

distilled and dried. Yield: 6.5 g, 58%. ^{1}H NMR (400 MHz, d_6-DMSO) δ 8.53 (d, J = 7.2 Hz, 1H), 8.47 (dd, J = 7.6, 3.3 Hz, 2H), 7.88 (t, J = 7.8 Hz, 1H), 7.76 (d, J = 8.0 Hz, 1H).

Synthesis of **7**. To a solution (15 ml, DMSO:H_2O = 7:3) of **6** (1.26 g, 3 mmol), $NaHCO_3$ (756 mg, 9 mmol) and NaHS (504 mg, 9 mmol) were added. The reaction mixture was stirred at room temperature overnight. Then the mixture was poured into 120 ml ice water to obtain a yellow precipitate, which was collected by vacuum filtration and dissolved with ethyl acetate (150 ml). The organic solution was washed with water (50 ml) and brine each once and dried by anhydrous sodium sulfate followed by evaporation under reduced pressure, yielding a yellow powder **7** (1.10 g, 93%). R_f (5% MeOH in CH_2Cl_2), 0.3. ^{1}H NMR (400 MHz, $CDCl_3$) δ 8.68 (d, J = 7.2 Hz, 1H), 8.63 (d, J = 8.3 Hz, 1H), 8.50 (d, J = 8.4 Hz, 1H), 7.83 (t, J = 7.8 Hz, 1H), 7.13 (d, J = 8.4 Hz, 1H), 5.66 – 5.54 (m, 1H), 5.38 (s, 2H), 4.70 (d, J = 5.0 Hz, 2H), 3.33 (d, J = 2.6 Hz, 2H), 3.22 – 3.08 (m, 2H), 2.12 (d, J = 11.3 Hz, 2H), 1.97 (s, 9H); ^{13}C NMR (101 MHz, d_6-DMSO) δ 156.8, 156.2, 146.9, 144.3, 125.7, 122.7, 121.5, 120.1, 115.3, 113.8, 111.0, 100.2, 100.0, 71.5, 19.8, 19.3. HRMS: calcd for $[M{-}Boc{+}H]^+$, 296.1394; found 296.1396.

Synthesis of **4**. Compound **4** was obtained by treatment of **7** (290 mg, 0.73 mmol) with TFA:CH_2Cl_2 (1:1) solution and used for synthesis of **2** without column purification.

Synthesis of probe **2**. **4** (74 mg, 0.25mmol) was dissolved by 6 ml anhydrous DMF, TEA (104 μl, 0.75 mmol) and 4-Chloro-7-nitrobenzofurazan (46 mg, 0.23 mmol) in DMF was added respectively drop by drop. The reaction mixture was stirred t room temperature for 2.5 h and then poured into 100 ml ice water, which was distilled with ethyl acetate and dried. The resulting residue was subjected to column chromatography on silica (0.5% MeOH in CH_2Cl_2), yielding a red solid **2** (34 mg, 37%). R_f (8% MeOH in CH_2Cl_2), 0.5. ^{1}H NMR (400 MHz, d_6-DMSO) δ 8.58 (d, J = 8.3 Hz, 1H), 8.47 (d, J = 9.1 Hz, 1H), 8.36 (d, J = 7.2 Hz, 1H), 8.14 (d, J = 8.4 Hz, 1H), 7.61 (t, J = 7.8 Hz, 1H), 6.81 (d, J = 8.4 Hz, 1H), 6.70 (d, J = 9.2 Hz, 1H), 5.51 – 5.37 (m, 1H), 4.93 (s, 2H), 3.80 (s, 2H), 3.68 (t, J = 12.4 Hz, 2H), 2.85 – 2.73 (m, 2H),

1.92 (d, J = 10.1 Hz, 2H); ^{13}C NMR (101 MHz, d_6-DMSO) δ 164.2, 163.3, 152.7, 145.1, 144.9, 144.7, 136.4, 134.1, 131.1, 129.7, 129.3, 124.0, 122.1, 120.7, 119.1, 108.2, 107.6, 103.4, 49.8, 48.7, 40.2, 39.9, 39.7, 39.5, 39.3, 39.1, 38.9, 27.8. HRMS: calcd for $[M+H]^+$, 459.1411; found 459.1399.

Absorption analyses

Absorption spectra were recorded at room temperature on a UV-3600 UV-VIS-NIR spectrophotometer (SHIMADZU, Japan). 1000 mM Stock solutions of Na_2S in degassed PBS buffer were used as H_2S source. For the time course experiment, probes (10 μM) in PBS buffer (pH = 7.4, 50 mM, 30% DMSO) were added with 1 mM or 2 mM Na_2S at room temperature, and the absorption was measured at different time points. All measurements were performed in a 3 ml corvette with 2 ml solution.

Determination of the detection limit

The fluorescence emission spectra of probes **1, 2** and **3** without Na_2S was measured by 10 times and the standard deviation of blank measurement was obtained. Then solution with probes (1 μM) was treated with Na_2S at different concentrations. A linear regression curve was then achieved according to the fluorescence intensity at 540 nm versus Na_2S concentrations. The detection limit was calculated with the following equation: Detection limit = 3σ/k. Where σ is the standard deviation of blank measurements, k is the slope between the fluorescence intensity at 540 nm versus Na_2S concentrations. The detection limit of probes **1**, **2** and **3** was determined to be 0.9 , 2.5 and 2.6 μM, respectively.

Calculation of fluorescence turn-on fold

To calculate the turn-on fold, we determined the emission spectra of the three probes in the absence and presence of H_2S. The fluorescent intensity change at 540 nm before and after reacting with H_2S was divided to give the turn-on fold.

MTT assay

HEK-293 cells were maintained in exponential growth, then seeded in 96 well plate at the density about 5000/well. There was also a background group with only medium in each well but no cells. When the confluence reached 90%, cells were treated with probe **1** at the final concentration from 1 μM to 10 μM, control groups were only treated with the same amount of DMSO. After incubation at 37 ℃, 5% CO_2 for 16 h, each well of cells were treated with 20 μl MTT solution (5 mg/ml), and incubated for another 4 h. Then each well was treated with 150 μl DMSO at 37 ºC for 10 min, and then the OD_{470} for each well were recorded on Safire 2. Cell viability was calculated by using the formula: (experiment group - background) / (blank group - background)*100%.

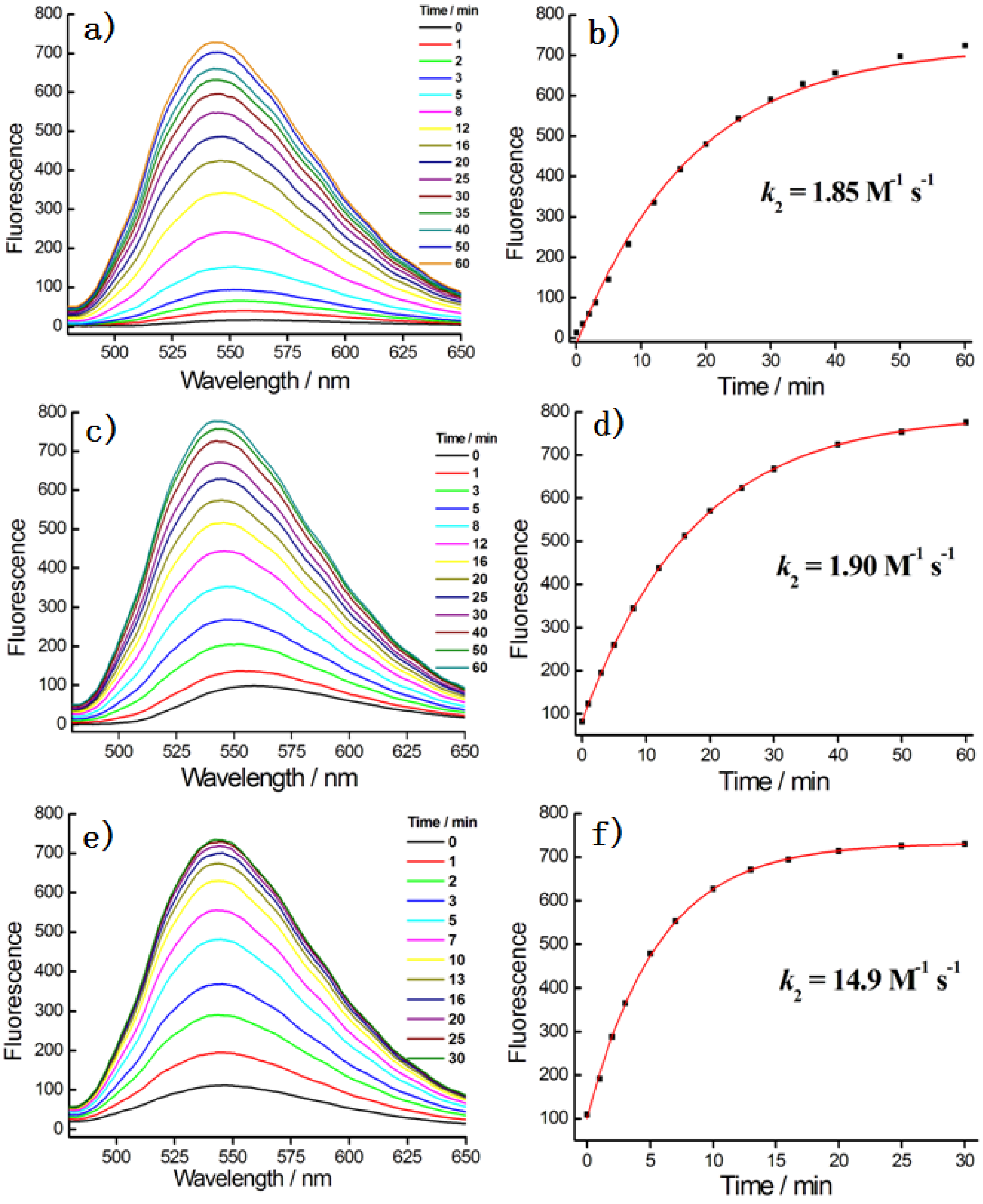

Figure S1. Time-dependent fluorescent spectra and dynamic simulation of probes 1-3 (a) Time-dependent fluorescent spectra of 1μM **1** with 500 μM H_2S; (b) dynamic simulation (the red solid line) of fluorescence intensity at 540 nm versus time from (a); (c) time-dependent fluorescent spectra of 1μM **2** with 500 μM H_2S; (d) dynamic simulation (the red solid line) of fluorescence intensity at 540 nm versus time from (c); (e) time-dependent fluorescent spectra of 1μM **3** with 200 μM H_2S; (f) dynamic simulation (the red solid line) of fluorescence intensity at 540 nm versus time from (e).

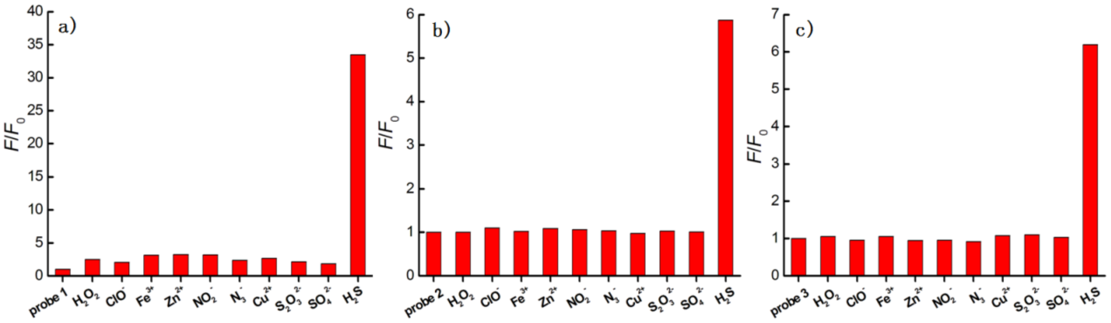

Figure S2. Fluorescent intensity at 540 nm for probes **1-3** (1 μM) toward different species in PBS buffer. (a, b) Reacrions of **1** and **2** with different species were performed for 60 min. (c) Reacrions of **3** with different species were performed for 20 min.The test lanes: H_2O_2 (200 μM), ClO^- (200 μM), Fe^{3+} (200 μM), Zn^{2+} (200 μM), NO_2^-(200μM), N_3^- (200μM), Cu_2^+ (200μM), $S_2O_3^{2-}$ (200 μM), SO_4^{2-} (200 μM), H_2S (200 μM).

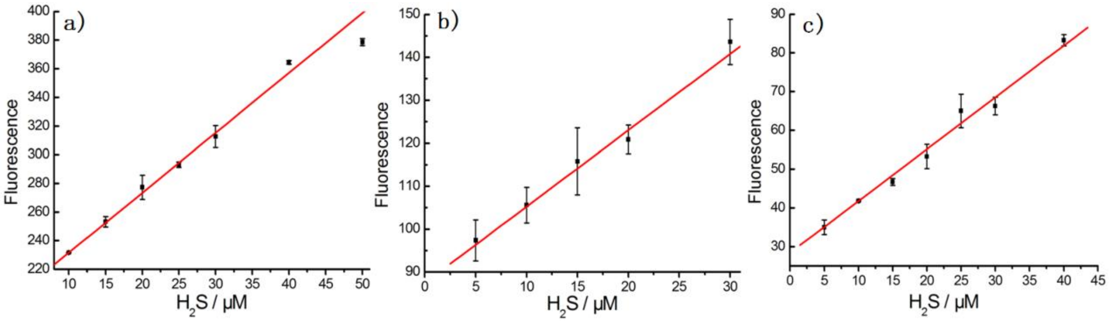

Figure S3. Linear relationship between fluorescence intensity at 540 nm of probes and H_2S concentrations. (a) Probe **1** (1 μM); (b) probe **2** (1 μM); (c) probe **3** (1 μM).

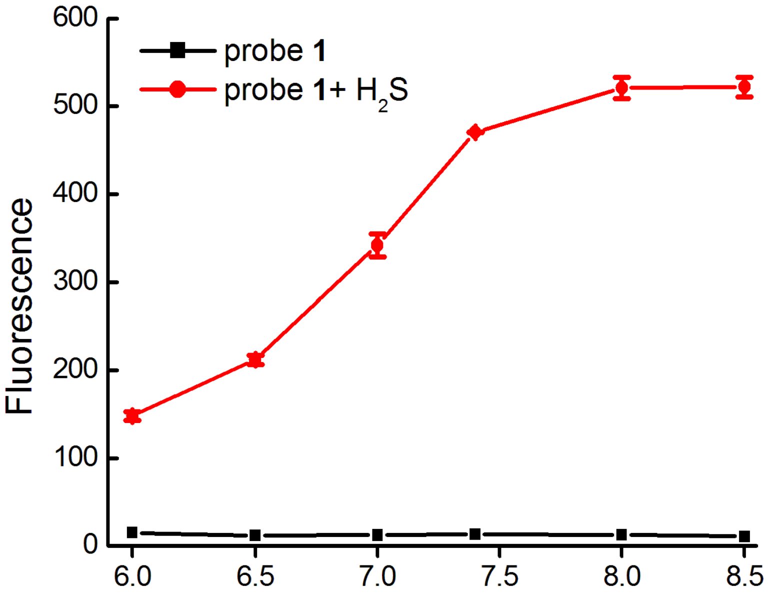

Figure S4. Emission at 540 nm of probe **1** (1 μM) at indicated pH values in the absence or presence of H_2S (200 μM).

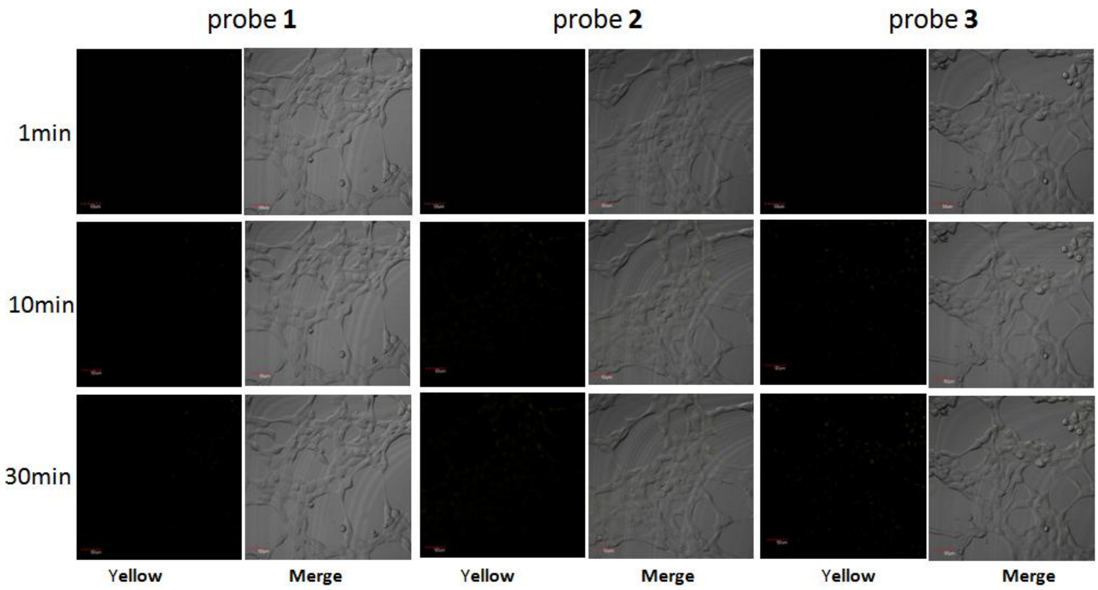

Figure S5. Confocal microscopy images of SO_3^{2-} in living cells using probes **1-3**. HEK293 cells were incubated with **1** (5 μM), **2** (5 μM), **3** (5 μM) for 30 min, washed by PBS buffer, and then incubated with SO_3^{2-} (250 μM) for 1, 10, 30 min respectively. Scale bar, 50 μm.

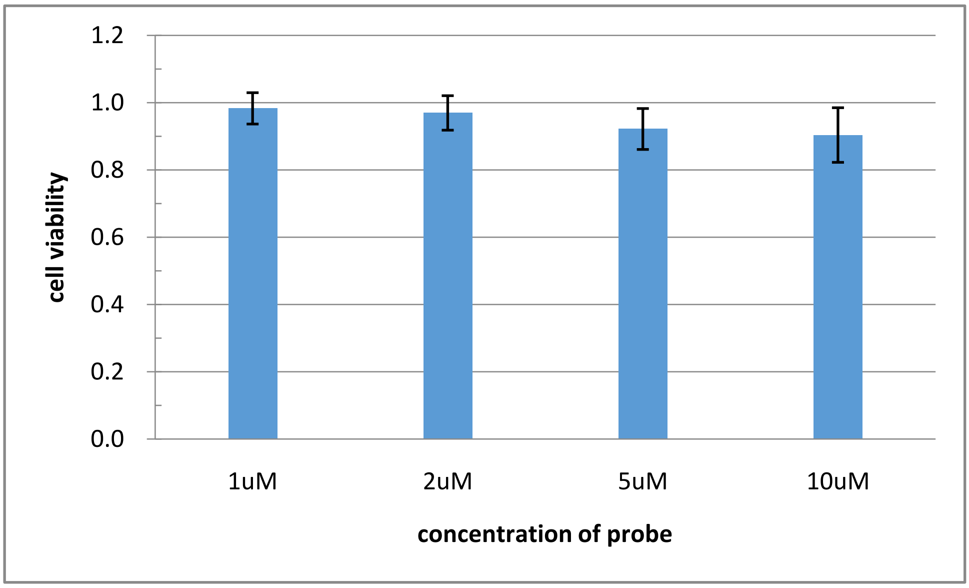

Figure S6. The cytotoxicity of probe **1** evaluated by the MTT assay.

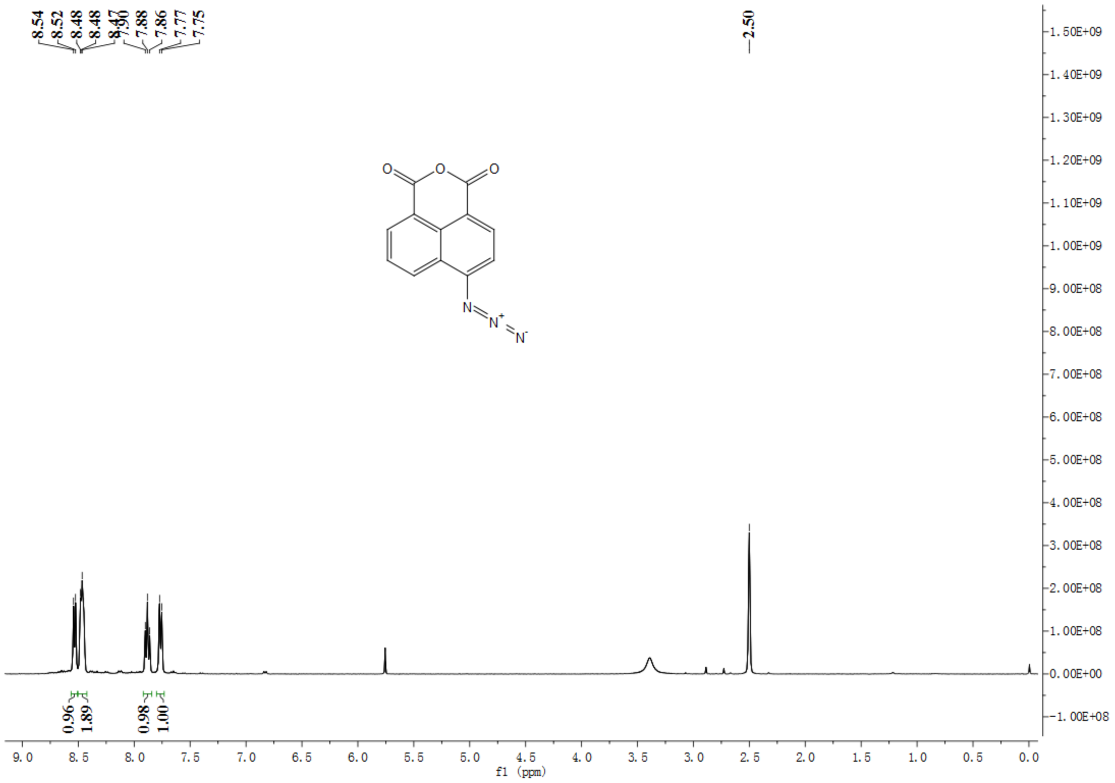

Figure S7. ^{1}H-NMR of compound **5**.

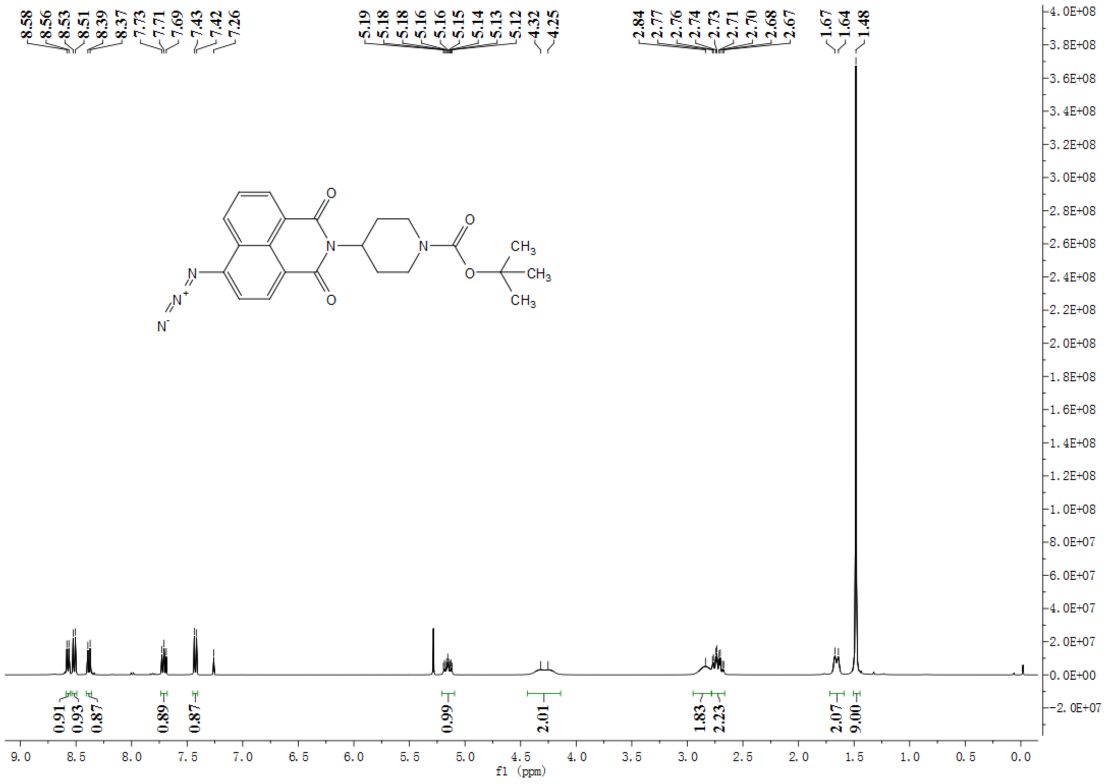

Figure S8. ^{1}H-NMR of compound **6**.

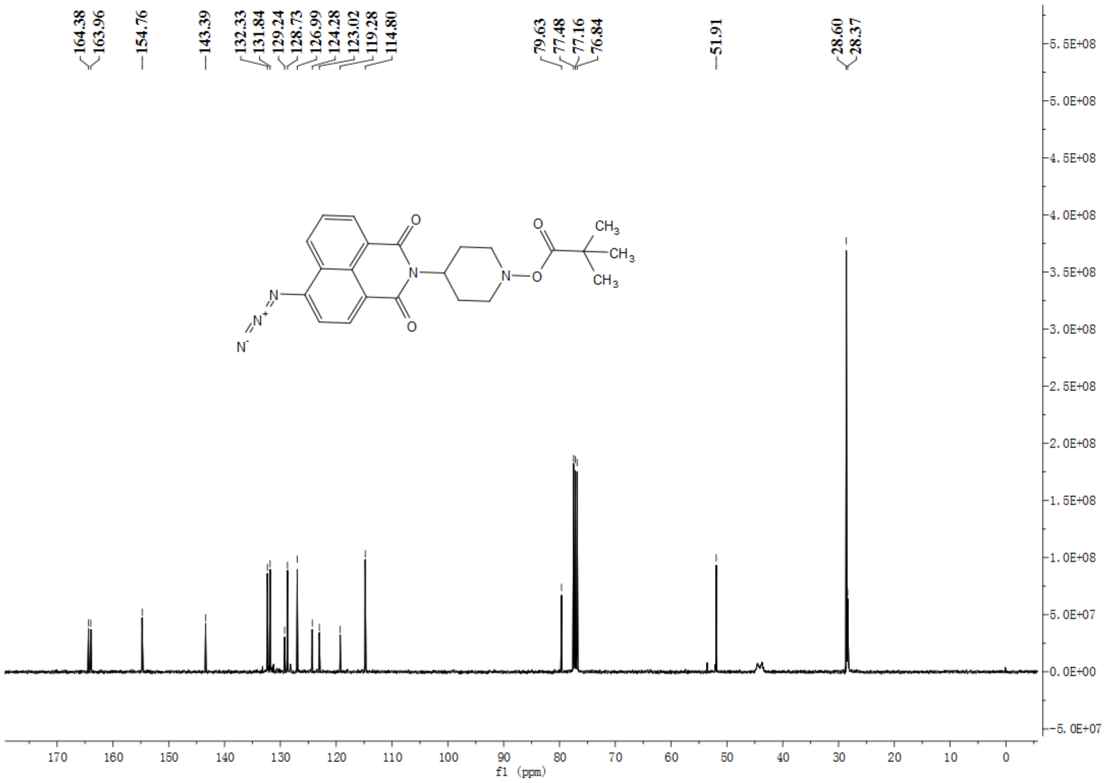

Figure S9. ^{13}C-NMR of compound **6**.

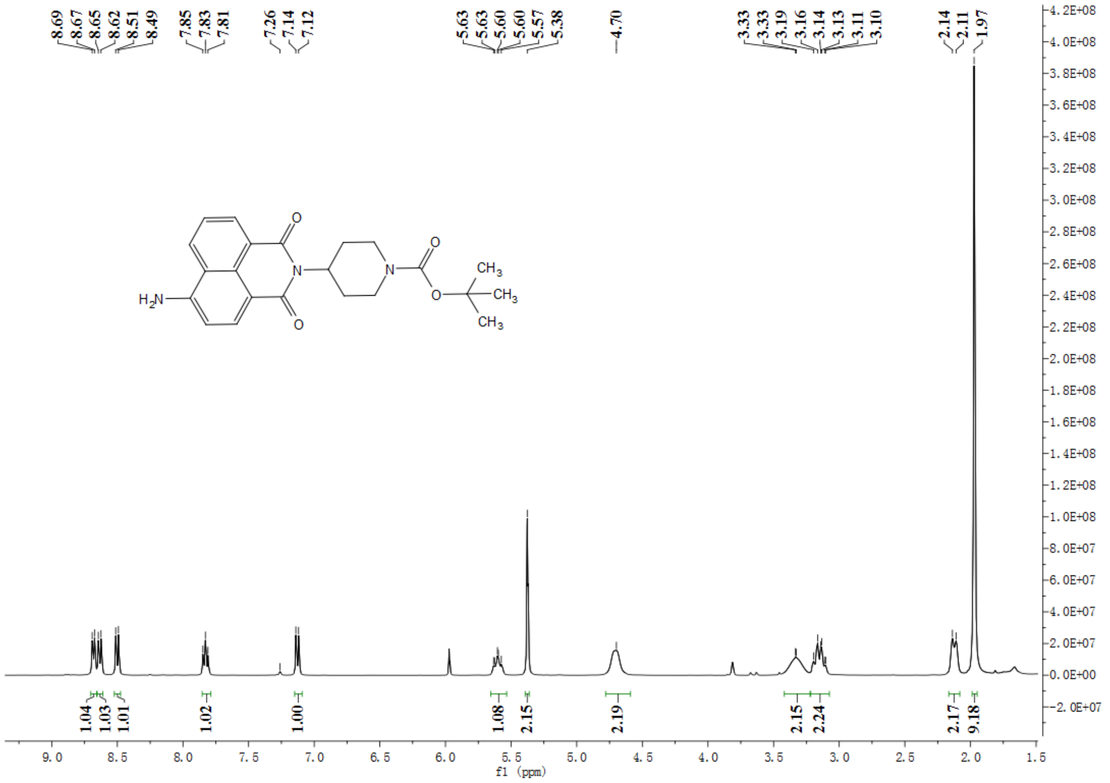

Figure S10. ^{1}H-NMR of compound **7**.

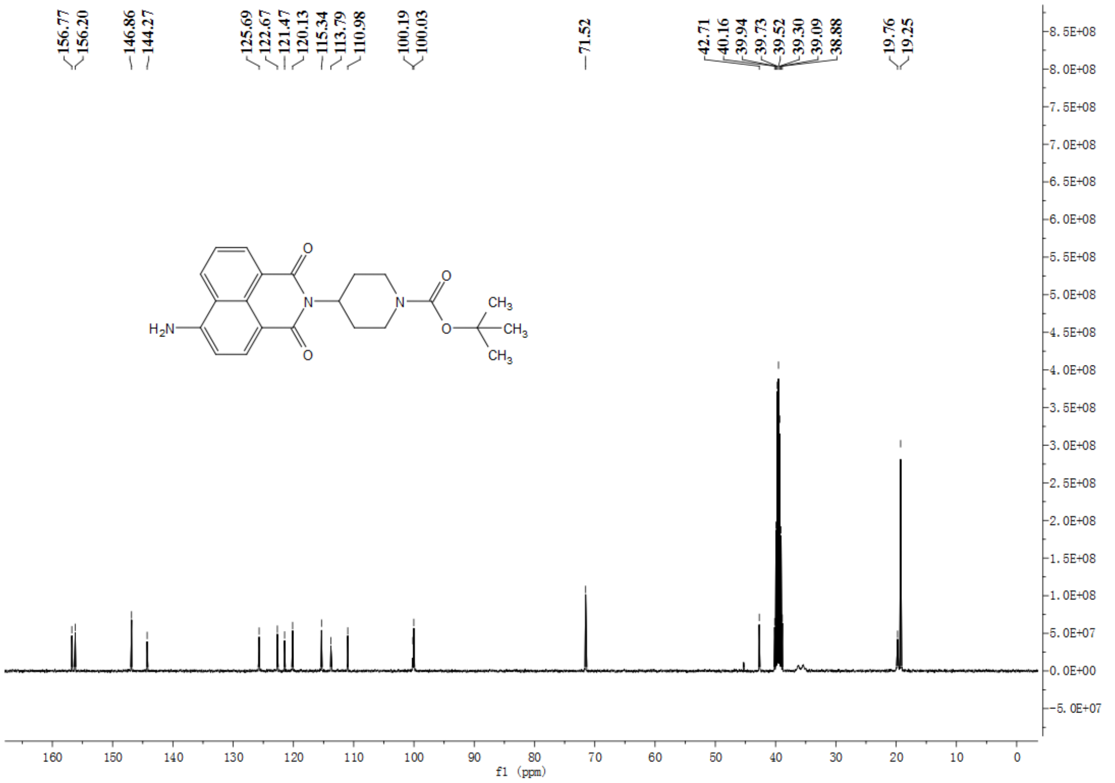

Figure S11. ^{13}C-NMR of compound **7**.

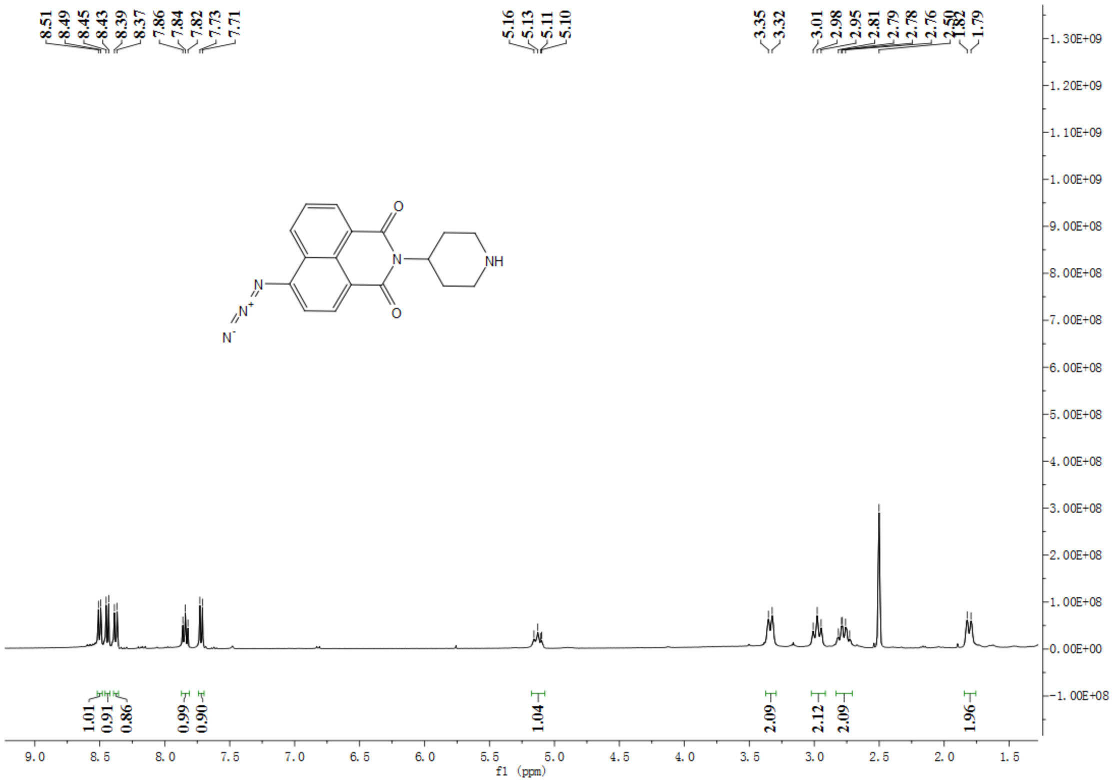

Figure S12. ^{1}H-NMR of probe **3**.

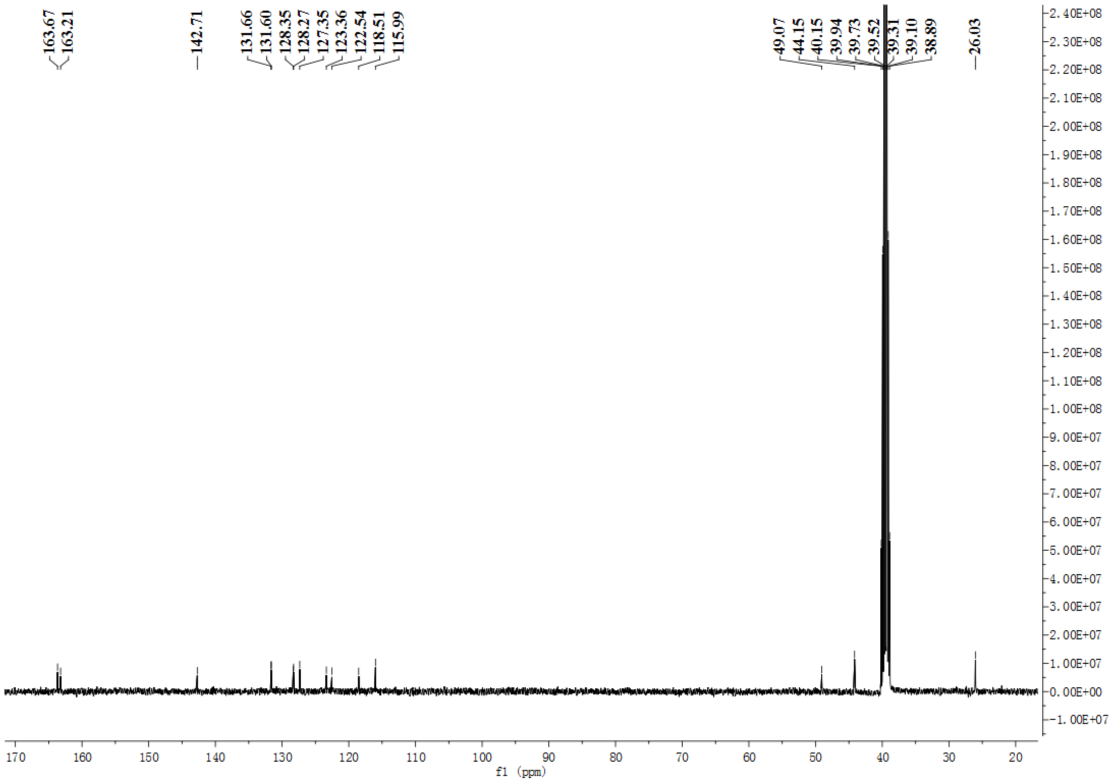

Figure S13. ^{13}C-NMR of probe **3**.

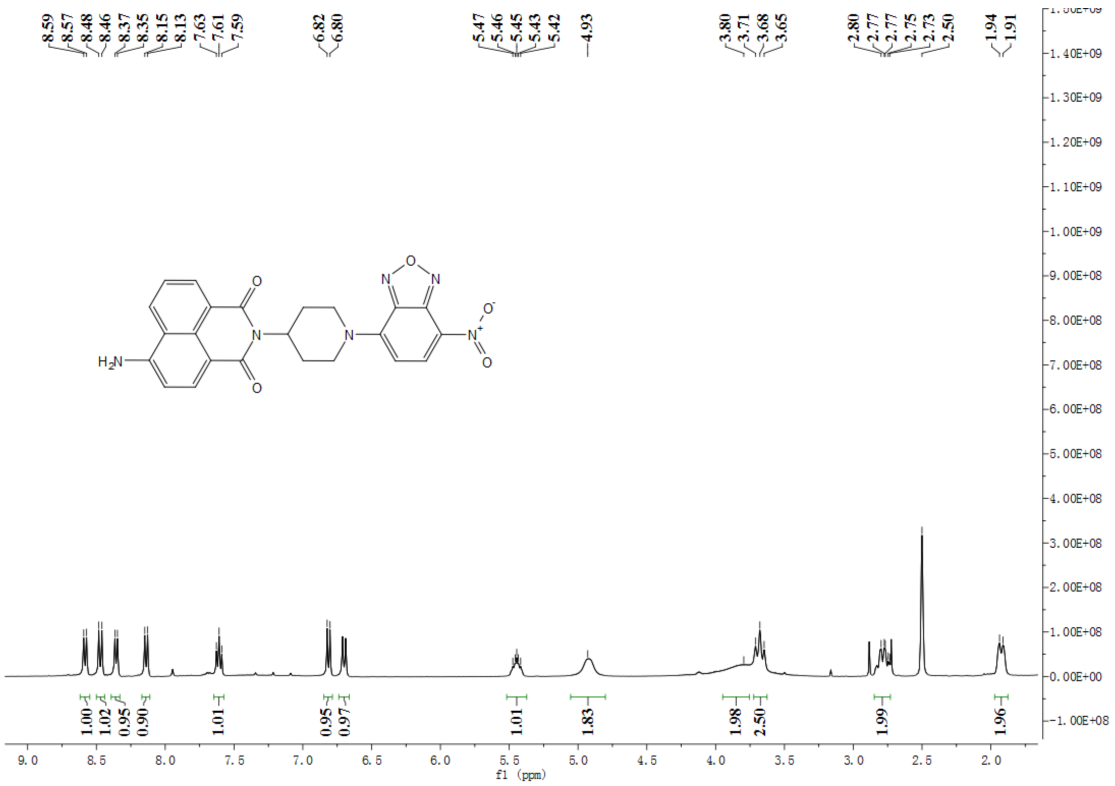

Figure S14. ^{1}H-NMR of probe **2**.

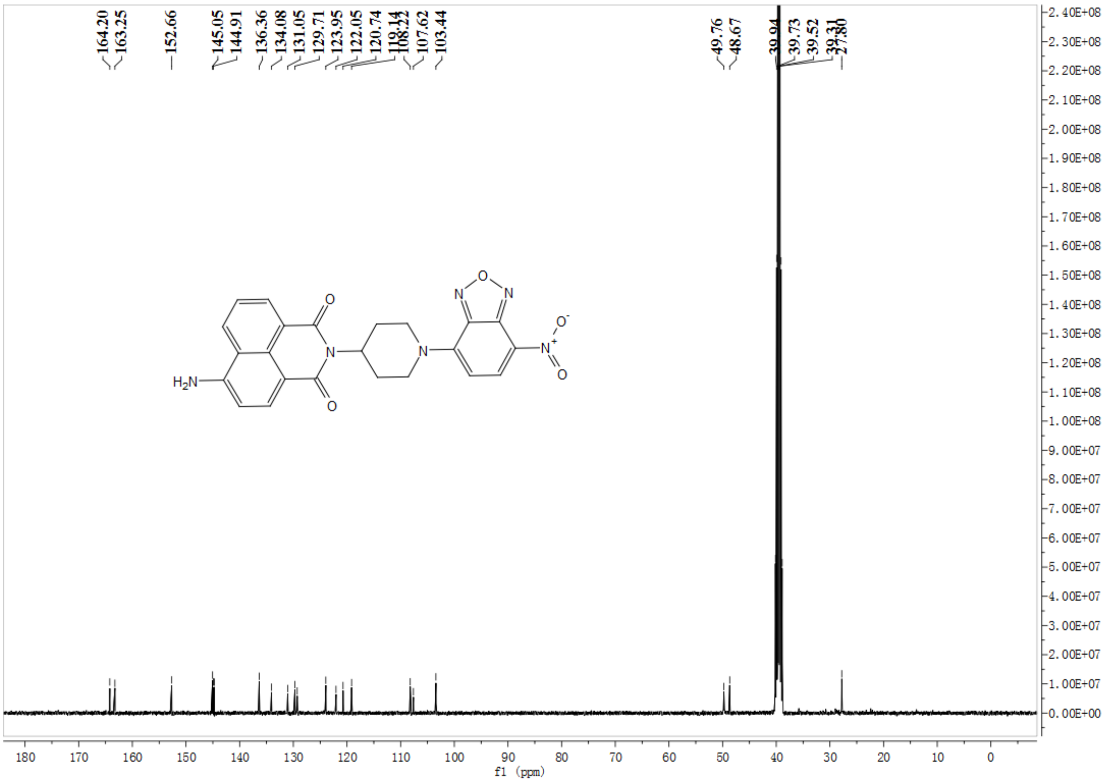

Figure S15. ^{13}C-NMR of probe **2**.

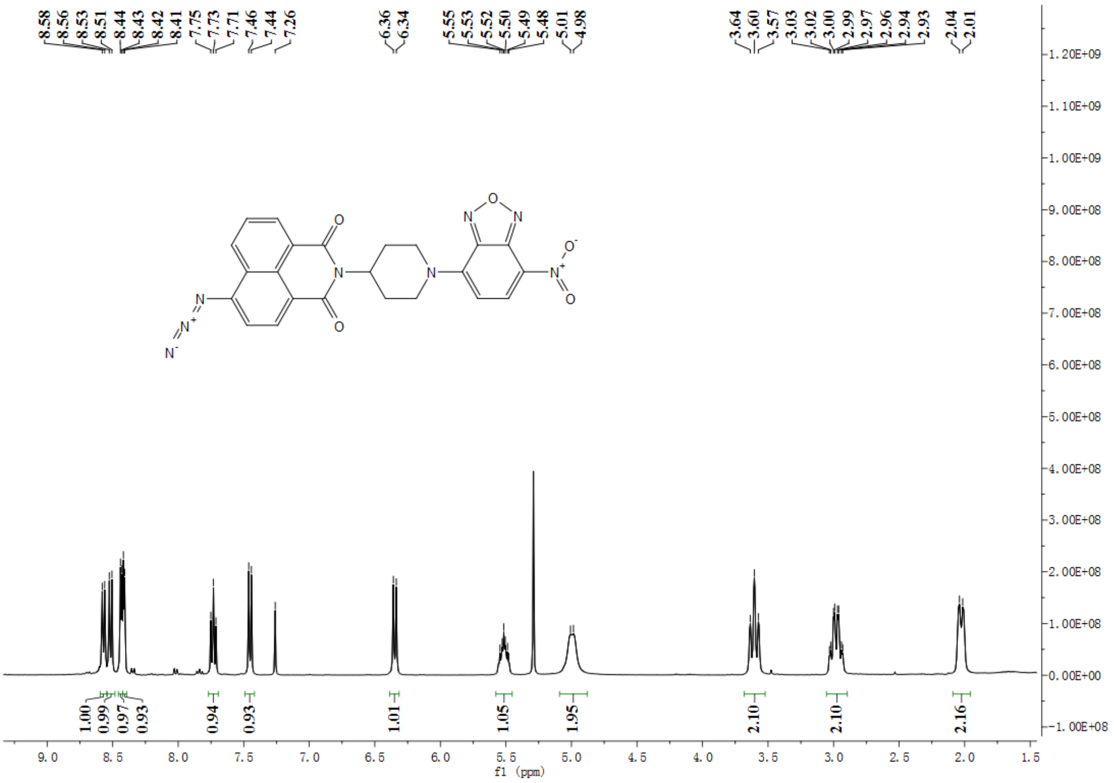

Figure S16. ^{1}H-NMR of probe **1**.

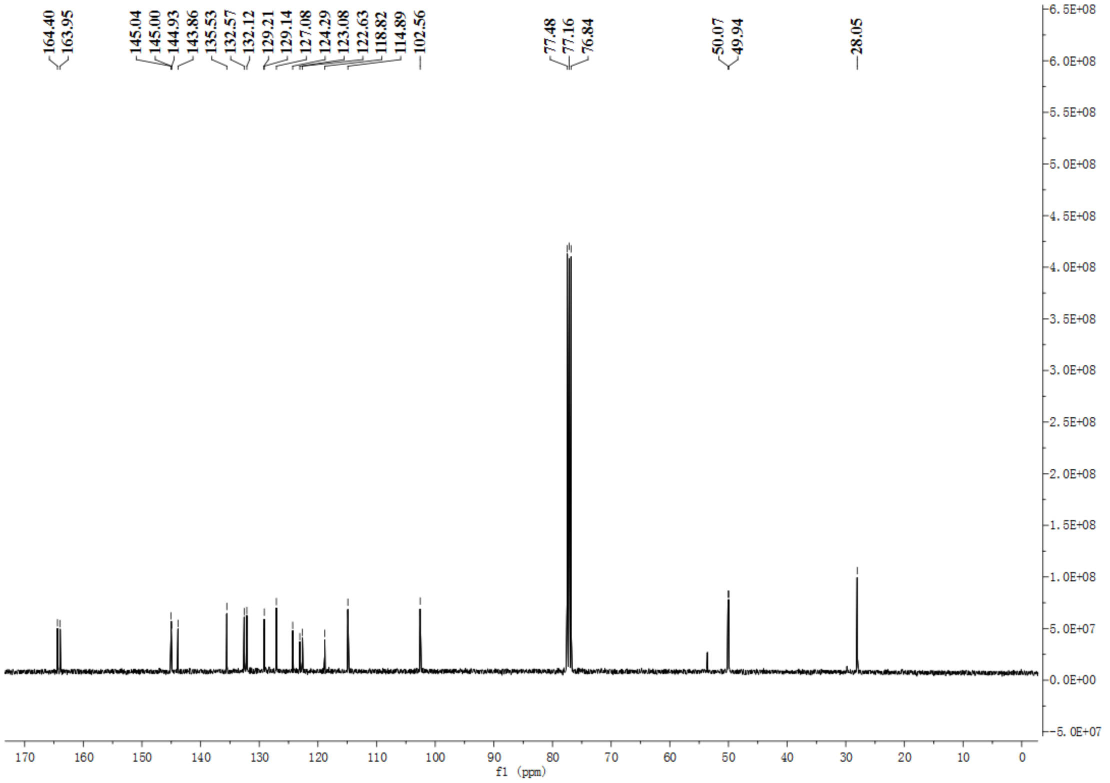

Figure S17. ^{13}C-NMR of probe **1**.

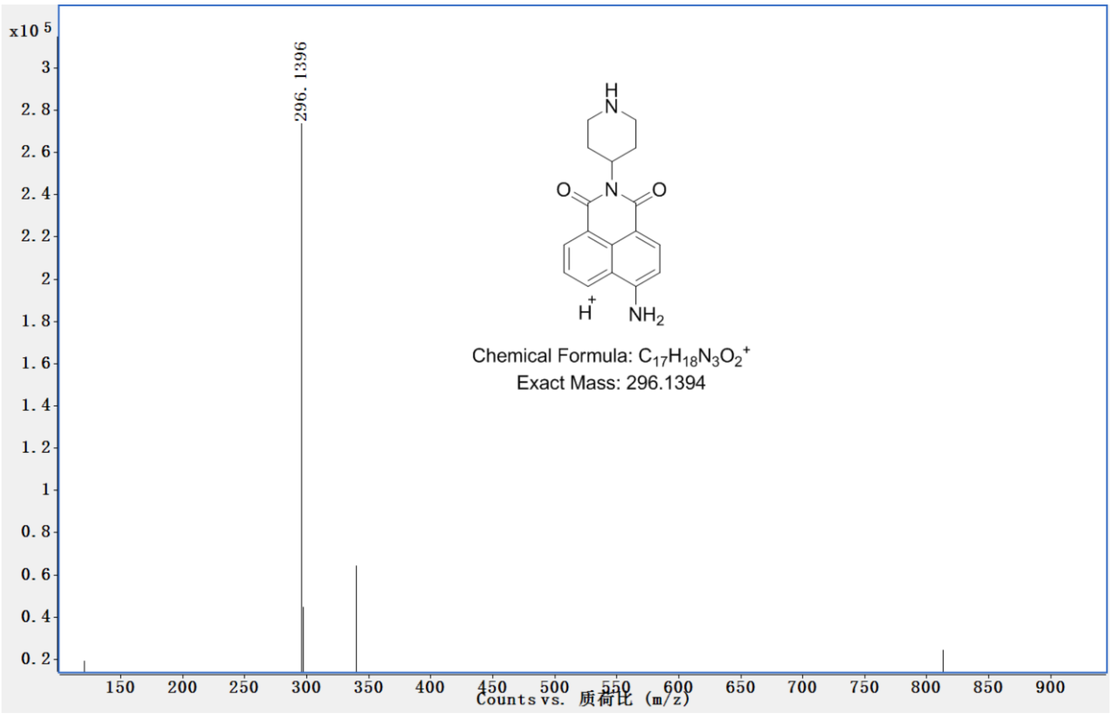

Figure S18. HRMS of compound **7**.

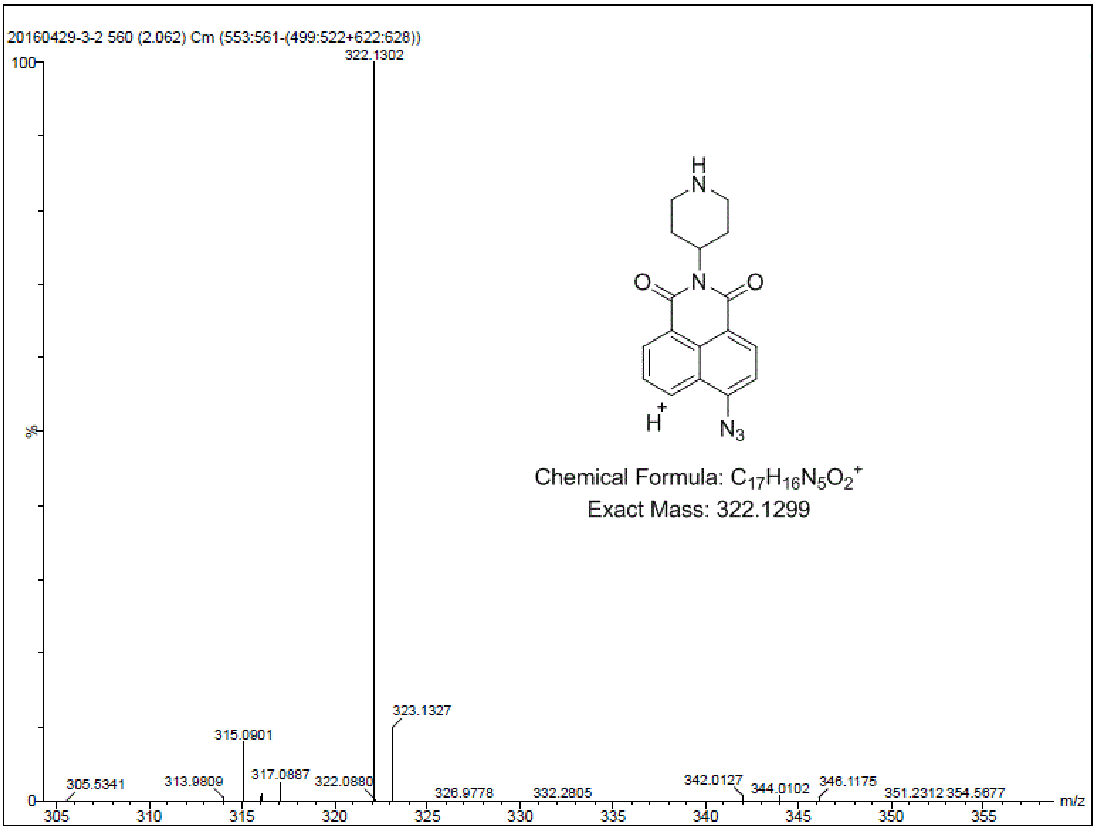

Figure S19. HRMS of probe **3**.

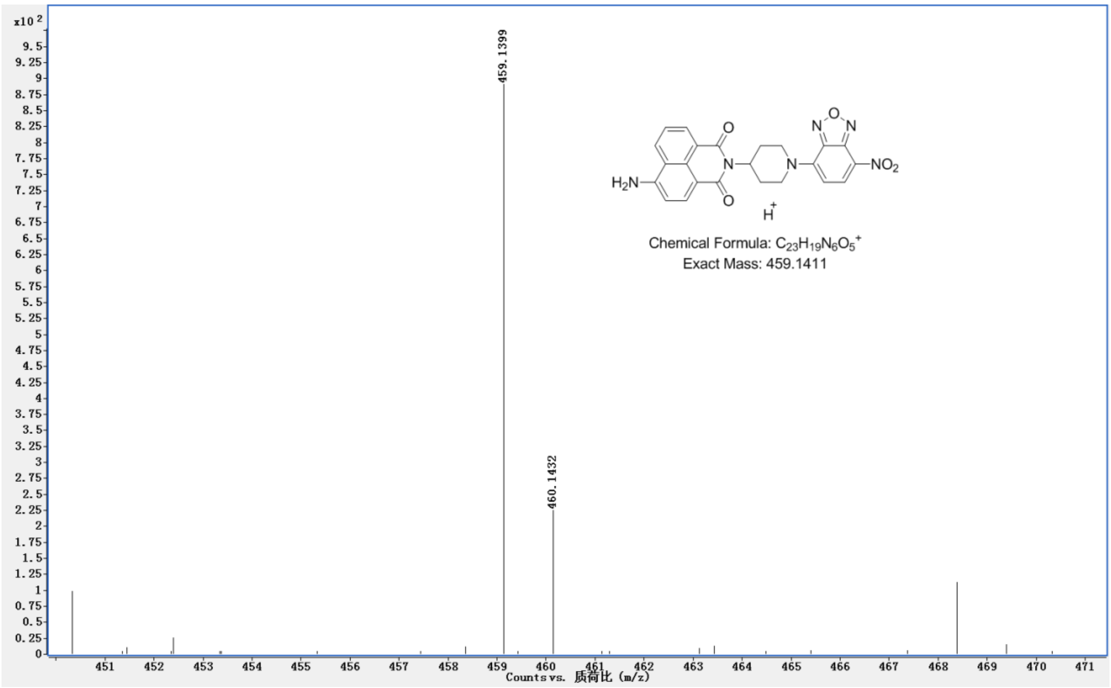

Figure S20. HRMS of probe **2**.

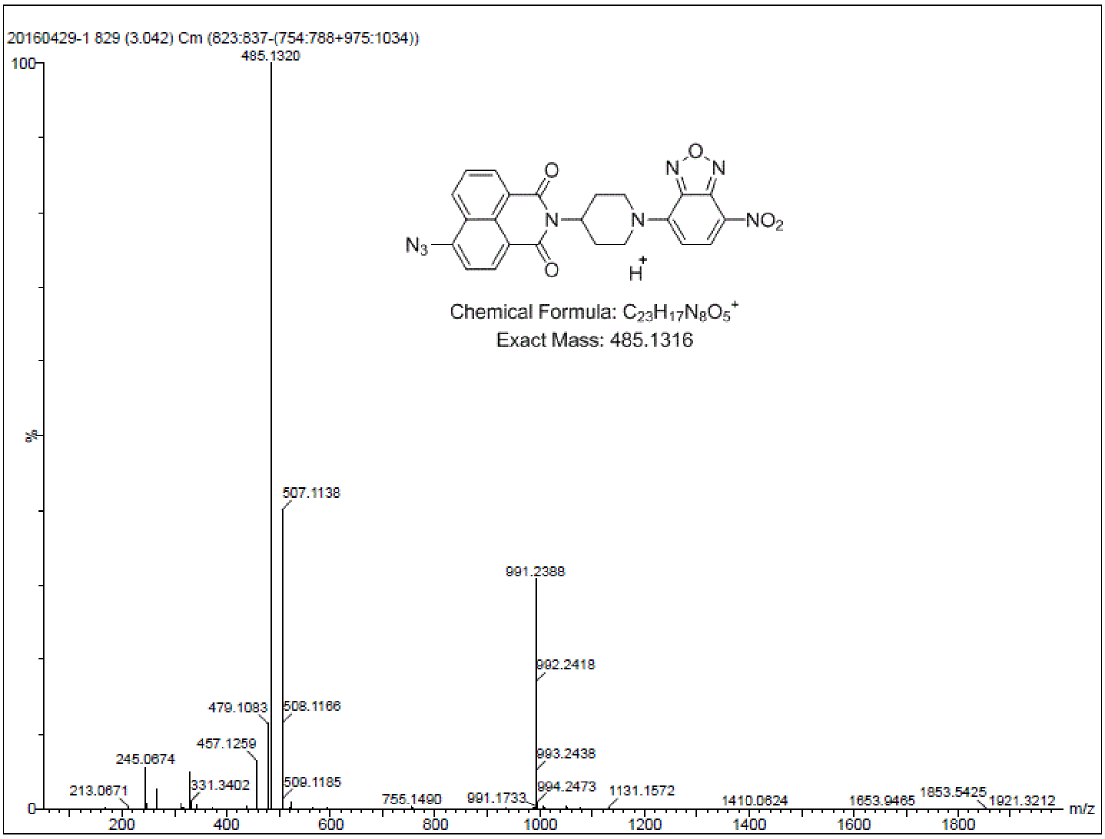

Figure S21. HRMS of probe **1**.
